# Comparing Laboratory and Synchrotron X-Ray CT for Structural Analysis of PEEK Orthopedic Implants

**DOI:** 10.3390/biomimetics11050357

**Published:** 2026-05-21

**Authors:** Meili Qi, Yanwei Zhao, Jinwen Chen, Shengtao Zhang, Jie Zhang, Xu Zhang

**Affiliations:** 1 School of Civil Engineering, Shandong Jiaotong University, Jinan 250357, China; qimeili@sdjtu.edu.cn (M.Q.); cjw051019@163.com (J.C.); zhangshengtao2005@163.com (S.Z.); 15379748955@163.com (J.Z.); 2 Shandong Key Laboratory of Technologies and Systems for Intelligent Construction Equipment, Shandong Jiaotong University, Jinan 250357, China; 3 National United Engineering Laboratory for Biomedical Material Modification, Dezhou 251100, China; zhaoyw@brandentech.com

**Keywords:** X-ray CT, biomedical PEEK implants, edge enhancement, orthopedic implant evaluation

## Abstract

Polyetheretherketone (PEEK) is widely employed in orthopedic applications due to its bone-mimetic mechanical properties and excellent biocompatibility, establishing it as a promising candidate for bone repair and regeneration. However, the investigation of structural integrity and microstructural features of PEEK implants has remained limited owing to its low atomic number (Z < 8) and hierarchical microstructure. This study presents a comparative characterization of PEEK implants by integrating laboratory X-ray computed tomography (CT) and synchrotron X-ray phase-contrast tomography (SXCT). Laboratory X-ray CT, operating on absorption contrast mechanisms, demonstrates adequate capacity for visualizing macroscopic defects and pore architectures, but exhibits limitations in resolving subtle density variations. In contrast, SXCT, employing phase-contrast imaging, significantly enhances discrimination of ultra-low-contrast features, including interconnected pore networks and localized density gradients. This work elucidates the complementary advantages and inherent limitations of both the imaging modalities for characterizing PEEK-based biomaterials. Furthermore, it demonstrates the potential extensibility of this comparative approach to other polymer composites, offering a methodology to investigate material–tissue interactions in orthopedic research and advancing the development of next-generation implantable devices.

## 1. Introduction

Pure PEEK is bioinert and acts primarily as a structural substitute; its bone-mimicking mechanical properties help reduce stress shielding compared to stiffer metallic implants. However, pristine PEEK does not actively promote bone regeneration. To impart osteoconductivity and osseointegration capability, PEEK must be suitably modified, for example, through surface sulfonation [1] or by incorporating bioactive fillers to form composites [2,3]. These modified PEEK formulations are the subject, and they illustrate how surface and bulk modifications can transform PEEK from a passive spacer into a pro-regenerative scaffold. By closely mimicking the mechanical attributes of bone, PEEK minimizes stress shielding, a condition leading to resorption or weakening of surrounding bone tissue, while providing robust support [4,5,6]. Additionally, its transparency to X-rays, computed tomography (CT), and magnetic resonance imaging facilitates effective postoperative monitoring, enabling clear assessment of healing and integration without interference from the implant material [7,8,9]. Despite these advantages, conducting detailed structural analyses of PEEK implants remains challenging due to their low atomic number (Z < 8) and complex multiscale microstructure [10,11,12]. The selection of imaging methods significantly influences the detail and accuracy of structural analysis, making it crucial to choose the most suitable technique based on specific research and clinical requirements [13,14,15,16,17]. To ensure the structural integrity and functional optimization of PEEK implants, non-destructive imaging techniques, such as X-ray CT, are a good choice. However, the choice of imaging modality, laboratory or synchrotron X-ray CT, critically influences the accuracy of microstructural characterization. Therefore, systematic evaluations of imaging techniques for assessing PEEK implant structural integrity and microstructural features is highly valuable.

On one hand, laboratory X-ray CT achieves a resolution of several tens to hundreds of microns (the actual smallest resolvable feature, quantifiable via the modulation transfer function) within the bioimaging field, significantly enhancing the accuracy of microstructural characterization of PEEK materials [18,19,20,21]. While laboratory X-ray CT enables rapid macroscopic evaluation, its reliance on absorption contrast limits its ability to resolve ultra-low-contrast features (e.g., interconnected pores) and introduces artifacts that compromise quantitative analysis. On the other hand, synchrotron X-ray CT overcomes these limitations by leveraging phase-contrast imaging, achieving sub-10 µm resolution and enhanced sensitivity to subtle density variations by converting phase shifts into intensity modulations, showing edge-enhanced visualization of weakly absorbing structures [22,23]. Phase-contrast imaging can significantly reduce common polychromatic X-ray artifacts in laboratory CT, notably beam hardening and scattering, which frequently cause non-uniform gray values and blurred interfaces in polymer-based materials. Synchrotron X-ray CT, with its high-throughput X-ray beam, achieves high-contrast and high-resolution imaging, bringing about significant advancements in the study of PEEK materials [24,25,26]. High-resolution structural insights obtained via synchrotron X-ray CT can guide the optimization of fabrication parameters (e.g., porogen size, pressing conditions). The resulting optimized porous architecture, for instance with controlled pore interconnectivity and wall roughness, has been shown to facilitate cellular infiltration and significantly improve in vivo osteoconductivity and osseointegration strength [27].

This work establishes a high-resolution imaging paradigm suitable for the preclinical structural optimization and quality control of next-generation porous PEEK implants. Also, recent studies highlight the importance of protocol optimization but lack a comparative analysis of modality-specific trade-offs in PEEK characterization [28,29]. Such trade-offs include the balance between spatial resolution and scan accessibility, or between image contrast and operational cost.

In this study, we evaluated absorption-based laboratory X-ray CT and synchrotron X-ray CT techniques for assessing the structural integrity of PEEK implants. Our work integrates the cost-effective macroscopic screening capability of laboratory X-ray CT with the high phase-sensitive analysis offered by synchrotron X-ray CT, creating a workflow that balances speed, cost, and precision. By leveraging laboratory X-ray CT for rapid triage and synchrotron CT for targeted high-fidelity imaging, our work maximizes information yield while minimizing resource utilization. Specifically, this study aims to compare laboratory and synchrotron CT in PEEK implant evaluation and demonstrate how hybrid imaging can enhance structural analysis to support PEEK implant development.

## 2. Materials and Methods

### 2.1. PEEK Fabrication

**Figure 1:**
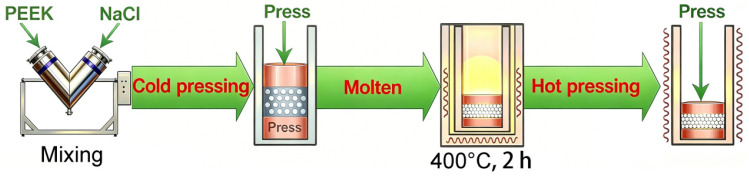
Experimental flow chart.

PEEK implant specimens were fabricated following the procedure detailed in our previous work [8,27]. In brief, as shown in Figure 1, medical-grade PEEK powder was mixed with a sacrificial porogen, cold pressed, and sintered to yield a porous architecture. The sintered specimens were then ultrasonically cleaned and dried. Complete processing parameters and characterization of the base material are available in [8,27].

### 2.2. PEEK Surface Morphology Characterization

Field emission scanning electron microscopy (FE-SEM, Zeiss, Oberkochen, Germany, Sigma 500) equipped with energy-dispersive X-ray spectroscopy (EDS) (Zeiss, Oberkochen, Germany.) mapping was used to characterize the surface morphology and elemental composition of the PEEK implants. Before observation, the samples were sputter-coated with a thin layer of gold to enhance conductivity.

A total of three independent PEEK implant specimens (*n* = 3) were fabricated and scanned using both laboratory CT and synchrotron CT. For quantitative comparison, ten equally spaced cross-sectional slices were selected per specimen, yielding 30 paired measurements for each quantitative metric (Pore size).

### 2.3. Laboratory X-Ray CT

**Figure 2:**
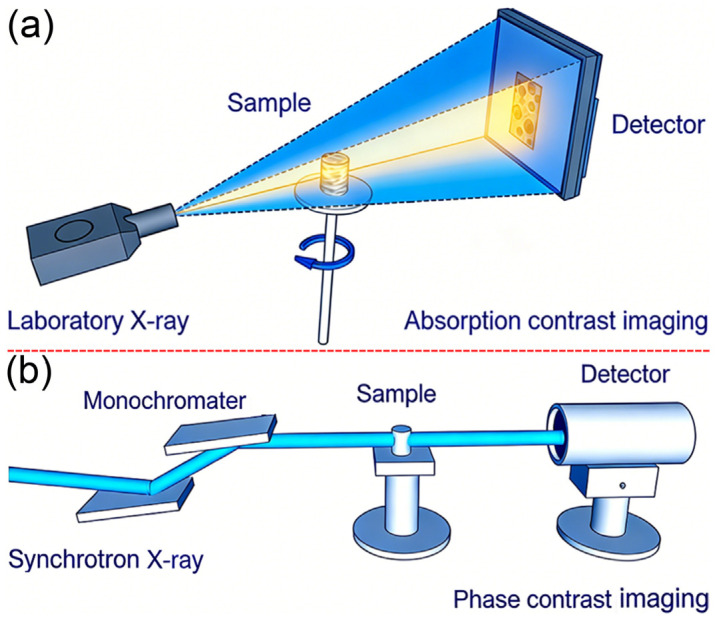
Schematic setup of laboratory X-ray CT (**a**) and synchrotron X-ray phase-contrast CT (**b**).

Laboratory X-ray CT characterization of PEEK implants was conducted using a CT system (SkyScan 2211) (Figure 2a). The laboratory-based X-ray microscopy operates on absorption contrast principles. Porous PEEK implants were scanned under the following conditions: a tube voltage of 50 kV, a tube current of 380 μA, and a 2 µm voxel resolution. A tube voltage of 50 kV was chosen because at this energy level, X-rays provide sufficient penetration power to clearly depict internal structures of PEEK materials. Additionally, this voltage range offers a higher signal-to-noise ratio (SNR), contributing to improved image quality. The 380 μA tube current was determined through a trade-off analysis between photon flux (for SNR optimization) and cumulative dose (for sample integrity and safety compliance). Considering the cumulative dose effect during prolonged scans, a lower tube current helps protect samples and minimize environmental impact. The CCD detector featured dimensions of 2688 × 4032 pixels. With a source-to-object distance (SOD) of 32.445 mm and a source-to-detector distance (SDD) of 146.882 mm, the magnification factor achieved was 4.5. Given the camera pixel size of 9.05 µm, this resulted in an effective image pixel size of 2 µm. To achieve a 2 µm resolution, 901 projections were acquired at 0.4° intervals (360° rotation), with a 950 ms exposure per projection, optimizing the angular sampling rate (0.4°) to minimize motion artifacts without excessive time investment. The chosen protocol results in a total scan time of approximately 0.6 h, which represents a practical compromise between image quality and throughput in a routine laboratory-based quality inspection setting. Increasing the number of projections from 1200 to 2400 with an exposure time of 1.0 s per frame extends the total scan time from approximately 0.6 h to 2.8 h. An exposure time of 950 ms per projection was determined based on preliminary experiments, providing adequate photon counts without adding significant noise, thus ensuring image clarity. The modified FDK algorithm in NRecon software (version 1.7.0) incorporated advanced ring artifact correction (10 iterations) and beam hardening compensation (20% polynomial fit), which are critical for mitigating artifacts in low-contrast PEEK materials with complex density gradients.

### 2.4. Synchrotron X-Ray Phase-Contrast CT

Synchrotron X-ray phase-contrast CT was performed at beamline BL13HB of SSRF (Figure 2b) [22]. The in-line phase-contrast X-ray microscopy setup utilized a double Si (111) crystal system to monochromatize the X-ray beam to an energy level of 10 keV. An energy of 15 keV was utilized due to its high coherence and penetration power, ideal for observing subtle density variations and edge enhancement effects. This is crucial for revealing minute pores and interface characteristics within PEEK implants. This optimization enhanced edge enhancement and transmission efficiency. As the X-ray beam traversed the specimen, it encoded both absorption and phase-shift data. After traveling a propagation distance of 20 cm, Fresnel diffraction transformed these phase shifts into detectable intensity variations, thereby achieving optimal image contrast between the sample and detector. By optimizing this distance, Fresnel diffraction mechanisms can be maximally exploited, significantly enhancing image contrast, thus allowing microscopic features smaller than 10 μm to become visible. The images captured combine absorption and phase-shift information using a CCD detector with a resolution of 2048 × 2048 pixels and a pixel size of 3.25 µm × 3.25 µm. The reconstructed volume had an isotropic voxel size of 3.25 µm, while the practical spatial resolution, estimated from the edge response at a sharp material interface, was approximately 6–8 µm. Contrast-to-noise ratio (CNR) was calculated using the standard pooled-variance formula: $\mathrm{CNR}=\left|\mu_{\mathrm{PEEK}}-\mu_{\mathrm{Background}}\right|/\sqrt{({\sigma_{\mathrm{PEEK}}}^{2}+{\sigma_{\mathrm{Background}}}^{2})/2}$ where S denotes the mean gray value and σ denotes the standard deviation measured in a uniform region of the PEEK material (avoiding visible pores, cracks, or inclusions) and in a homogeneous background region, respectively. Higher CNR indicates clearer visualization of low-contrast structures, critical for defect detection in CT. Pore connectivity was quantified as the ratio of the volume of the largest interconnected pore network to the total pore volume. Pore segmentation was performed using a global threshold determined by the Otsu method, and the connected component analysis was carried out using the Amira software (version 2021.1; Thermo Fisher Scientific, Waltham, MA, USA).

### 2.5. Quantitative Comparison of Laboratory-Based and Synchrotron CT Analyses

The sample was mounted directly onto a rotary stage precisely aligned with the CCD detector. During data acquisition, the stage rotated through 180 degrees, capturing 1800 projections from 0° to 180° at intervals of 0.01°, each with an exposure time of 50 milliseconds per projection. To account for temporal drift in detector sensitivity and X-ray flux stability, flat-field images were acquired every 100 projections, while dark-field images were captured pre-/post-scan to eliminate fixed-pattern noise. Tomographic reconstruction employed software specifically developed for the SSRF end station, incorporating background correction, alignment of the rotation axis, and filtered back-projection (FBP) reconstruction [27]. Three-dimensional rendering of the specimens was achieved using Amira software, allowing for detailed visualization and analysis of the internal structure of the PEEK samples.

## 3. Results and Discussion

### 3.1. Surface Morphology and Elemental Composition

**Figure 3:**
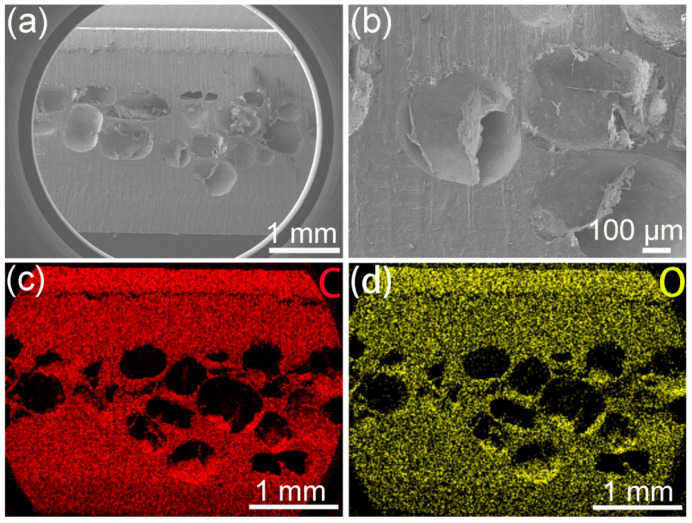
FE-SEM images and EDS mapping of PEEK implants. (**a**) Cross-sectional view, (**b**) porous layer detail, and (**c**,**d**) C and O element maps.

SEM imaging confirmed that all specimens exhibited a highly interconnected macro porous network, with pore diameters predominantly in the 200–500 µm range (Figure 3). This porous architecture provides the baseline structural framework for the CT-based comparisons that follow.

The observed macro porous network (Figure 3), with pore diameters predominantly in the 200–500 µm range and an overall porosity of approximately 65%, is designed to mimic the topological features of human cancellous bone. Cancellous bone typically exhibits pore sizes of 200–500 µm and a porosity of 50–90% [30]. This structural mimicry is critical because interconnected pores exceeding 100 µm are known to facilitate cell migration, nutrient transport, and vascularization, all prerequisites for bone ingrowth and long-term implant fixation.

### 3.2. Comparative CT Imaging of PEEK Implants

**Figure 4:**
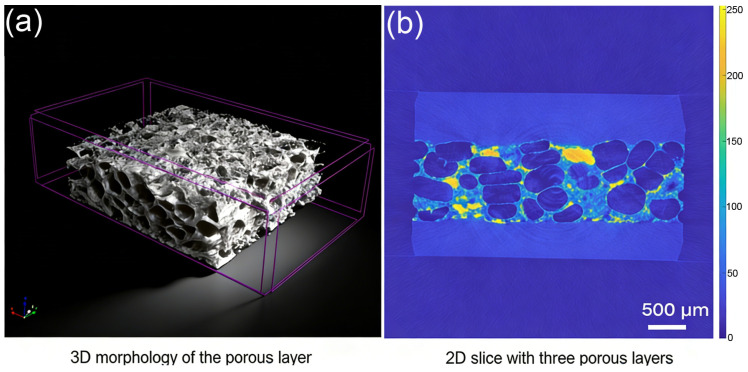
Laboratory X-ray CT imaging of PEEK implants. (**a**) 3D morphological reconstruction. (**b**) Representative 2D slice through the center.

While SEM provides detailed information on the surface morphology and elemental composition of the PEEK implants, it is inherently limited to two-dimensional surface observations. In contrast, CT enables non-destructive three-dimensional visualization of the internal structure, which is essential for evaluating the porous architecture and defects within the bulk material. Therefore, CT imaging was performed to complement the surface analyses. This study evaluated laboratory-based and synchrotron X-ray CT techniques for assessing the structural integrity of PEEK implants, establishing a comparative framework to guide biomedical research and clinical applications. The effectiveness of these imaging techniques in analyzing PEEK implants is largely determined by their spatial resolution and contrast capabilities. Laboratory X-ray CT achieves a pixel resolution of 2 µm, which is sufficient for routine applications. However, under the applied parameters (50 kV, 380 μA), it exhibits limited sensitivity to density gradients below 10 µm. Following CT reconstruction, the projections are converted into three-dimensional representations of the PEEK implants (Figure 4a), which can be virtually sectioned. A representative 2D slice through the center (Figure 4b) showcases the internal microstructure of the porous layer, highlighting structural heterogeneity, including pore size variations and potential defects.

**Figure 5:**
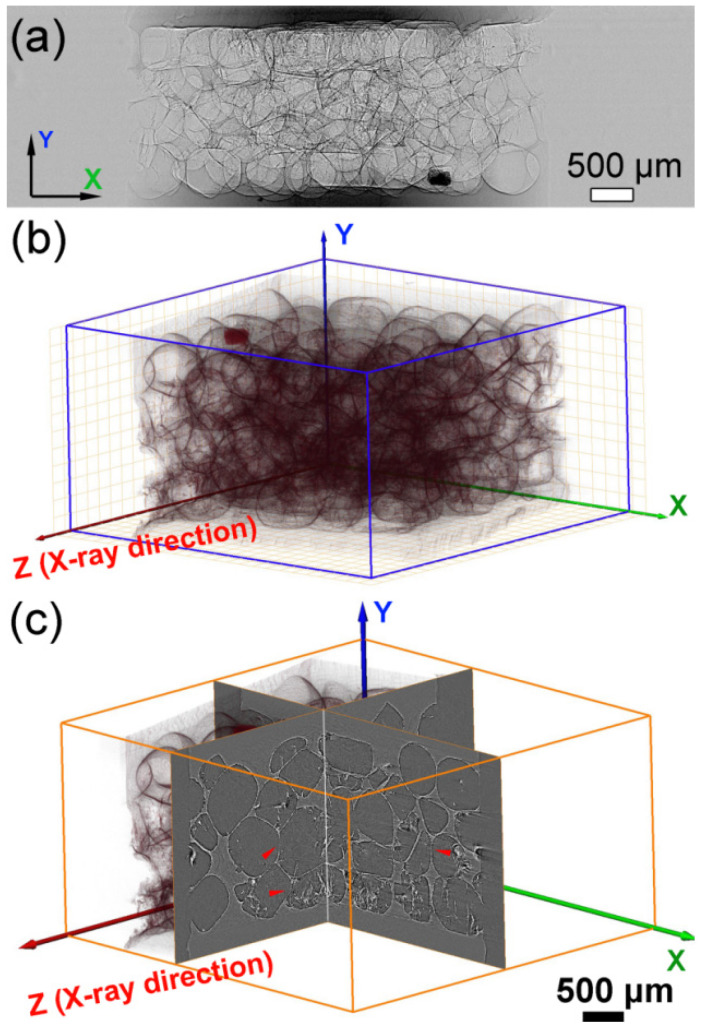
Synchrotron X-ray CT imaging of PEEK implants. (**a**) 2D projection image. (**b**) 3D morphological reconstruction. (**c**) Tomographic slice analysis showing sandwich-like structure and pore connectivity.

In contrast, synchrotron X-ray phase-contrast CT utilizes a 10 keV monochromatic beam and a propagation distance of 20 cm to exploit Fresnel diffraction, allowing for the detection of features below 10 µm (Figure 5a,b) and achieving a higher contrast-to-noise ratio (CNR) compared to laboratory X-ray CT. The 2D projection image (Figure 5a) reveals external morphology and provides initial insights into internal structures with enhanced contrast, demonstrating superior sensitivity in detecting subtle density variations. The 3D morphological reconstruction (Figure 5b) illustrates the intricate internal architecture of the PEEK sample. This improvement is critical for resolving subtle microstructural features, such as interconnected pores and localized density gradients. Across three independent samples, synchrotron CT detected a pore connectivity of 68% ± 5%, significantly higher than the 32% ± 8% observed with laboratory X-ray CT. Additionally, synchrotron CT revealed microcracks (<5 µm in width) near the interface between the dense and porous layers, and tomographic slice analysis (Figure 5c) clearly shows the sandwich-like structure and connectivity of the porous material (red arrows).

Quantitative CNR analysis was performed on 10 equally spaced slices from each of the three specimens (n = 30 per modality). The laboratory CT yielded a CNR of 3.2 ± 0.8, whereas the synchrotron CT achieved a CNR of 8.5 ± 1.9, representing a statistically significant improvement of approximately 165% (*p* < 0.001, paired *t* test).

Laboratory X-ray CT and synchrotron X-ray phase-contrast CT show markedly different capabilities in resolving the internal structure of PEEK implants. Laboratory systems (e.g., SkyScan 2211) use polychromatic X-ray sources (50 kV, 380 µA) and fixed magnification (4.5×), which limits energy tunability but enables long scans (43 min for 901 projections). In contrast, synchrotron facilities (e.g., BL13HB@SSRF) employ monochromatic beams (10 keV, ΔE/E ≈ 10^−4^) and adjustable propagation distances (0–50 cm), allowing precise control of imaging parameters, including switching between absorption and phase-contrast modes. Synchrotron CT also offers shorter scan time (10 min for 1800 projections) and higher flux density (10^9^ photons/s/mm^2^).

Quantitative analysis reveals that synchrotron X-ray CT achieves a 165% improvement in contrast-to-noise ratio (CNR) compared to laboratory CT, corresponding to a ~60% reduction in the minimum detectable defect size. This enables detection of microcracks (<5 µm width) at the dense/porous interface and resolves pore connectivity (68% ± 5% vs. 32% ± 8% for laboratory CT). These capabilities directly address the critical defect size range for implant failure under cyclic loading [19], overcoming prior limitations of laboratory CT in resolving sub-500 µm defects [13].

Regarding practical applicability, laboratory X-ray CT is widely available, cost-effective ($50–$100/scan), and compatible with clinical workflows, making it suitable for routine quality control. Synchrotron facilities offer superior precision but require beamtime proposals (success rate ~20–30% at SSRF) and incur higher costs (~$500–$1000/h), restricting their use to hypothesis-driven research. A balanced approach is therefore recommended: use laboratory CT for initial screening, and reserve synchrotron imaging for detailed analysis in complex cases.

The static imaging employed here does not capture dynamic processes such as fluid infiltration or mechanical loading; future studies integrating in situ testing could provide more physiologically relevant evaluations. Technological advances (e.g., iterative reconstruction algorithms, AI-driven segmentation) may help bridge the resolution gap for laboratory CT, while higher flux and optimized protocols will further enhance synchrotron CT. A pragmatic strategy leveraging the complementary strengths of both techniques will be essential as PEEK implant designs become more patient-specific and multifunctional.

It is important to consider that high-flux synchrotron X rays can potentially induce dose-dependent radiation damage in polymer samples, such as chain scission, discoloration, or local heating. In the present study, repeated scans of identical regions and careful visual inspection did not reveal any discernible structural or morphological changes in the PEEK specimens under the employed scan conditions. However, for more radiation-sensitive polymeric biomaterials, future studies are advised to employ mitigation strategies, including minimizing exposure time, using high frame rate detectors to lower per frame dose, or performing dose-fractionated scans. This consideration does not detract from the advantages of synchrotron phase-contrast imaging, but should be evaluated case by case when planning experiments on polymer-based implants.

## 4. Conclusions

In this study, we have presented a comparison of laboratory absorption-based CT and synchrotron phase-contrast CT for the 3D characterization of porous PEEK implants. Synchrotron imaging provided substantially higher CNR and revealed critical structural details, such as microcracks and pore wall topography, that were not reliably visualized with laboratory CT. This demonstrates that synchrotron-based phase-contrast imaging constitutes a powerful preclinical tool for the structural optimization and quality inspection of next-generation porous implants. The high-fidelity 3D datasets generated here can serve as direct input for future preclinical studies, including image-based finite element analysis (FEA) and ex vivo assessment of implant–tissue interactions in animal models. While the present study provides validated, quantitative 3D structural data ready for direct use in mechanical simulations, performing full finite element analysis (e.g., ANSYS) to predict implant stiffness and strength under physiological loading is beyond the current scope. This important integration of imaging with computational mechanics is a clear direction for our future work. While the imaging protocols described are not intended for routine clinical use or longitudinal patient monitoring, the structural insights they provide can inform the design of improved implants that ultimately translate into better clinical outcomes. Further work will focus on integrating this imaging workflow with mechanical and biological validations to establish a complete preclinical evaluation pipeline.

## Data Availability

Data are available from the corresponding author on reasonable request.
